# The prevalence of *Mycoplasma pneumoniae* in children in Shandong, China before, during, and after COVID-19

**DOI:** 10.3389/fped.2024.1479311

**Published:** 2024-12-11

**Authors:** Wenna Kong, Qianqian Wang, Jinhua Zhuo, Xuewei Zhuang

**Affiliations:** Department of Clinical Laboratory, Shandong Provincial Third Hospital, Shandong University, Jinan, Shandong, China

**Keywords:** *Mycoplasma pneumoniae*, COVID-19, children, epidemiological study, China

## Abstract

**Background:**

The multifaceted non-pharmaceutical interventions after the outbreak of the COVID-19 pandemic not only limited the spread of SARS-CoV2 but also had an impact on the prevalence of other pathogens.

**Methods:**

In this work, we retrospectively analyzed the epidemiological characteristics of *Mycoplasma pneumoniae* (MP) in children before and after the COVID-19 pandemic in Shandong, China. From 2019–2023, there were 29,558 visits of pediatric patients (1 month to 15 years old) with respiratory tract infection (RTI) symptoms at a tertiary hospital in Shandong Province, 10,039 of which were positive for MP according to a passive agglutination assay of the serum IgM antibodies. Conduct statistical analysis and epidemiological investigation of the test results categorized by years, months, ages, genders and clinical diagnosis. Utilize the *χ*^2^ test to analyze the differences in incidence rates.

**Results:**

Compared to 2019, the number of visits and the positive cases both decreased substantially in 2020, but the positivity rate increased. Both 2021 and 2023 were peak years of MP infection. The peak seasons of MP infection were fall and winter, female patients had higher positivity rate than male patients, and school-age children (>6 years) had higher positivity rate than the children in other age groups. In terms of the clinical manifestation of MP infection, compared to 2019, in 2023, the proportion of bronchopneumonia and upper RTI decreased significantly.

**Conclusions:**

The ongoing surveillance of the epidemiology of MP is critical for effective disease management and provides a basis for diagnosis, treatment, and the corresponding prevention and control strategies. This work for the first time characterized the epidemiology of MP in Shandong before and after the COVID-19 pandemic, thus providing valuable information for monitoring and preventing MP infection in the post-epidemic era.

## Introduction

*Mycoplasma pneumoniae* (MP) is an important pathogen that causes acute upper and lower respiratory tract infections (RTIs) as well as extrapulmonary manifestations. It accounts for up to 40% of the community-acquired pneumonia (CAP) in children, especially among those of school age (1). Refractory MP pneumonia can be life-threatening, especially for infants and the elderly. The epidemiological characteristics of MP infection vary by geographic region, age, sex, etc., and seasonality also exists. MP infection has a cyclic nature, and its incidence has been rising worldwide since 2010 (2). An epidemic of MP occurred in 2015–2016 in Beijing, China and in England, (1, 3), and in the US, a reemergence of MP infections in children and adolescents after the COVID-19 pandemic has been reported (4).

COVID-19 is a highly contagious disease caused by the severe acute respiratory syndrome coronavirus 2 (SARS-CoV-2) and it may lead to life-threatening complications. To prevent the spread of COVID-19, China adopted strict public health measures including mask mandates, social distancing, limits on gatherings and outdoor activities, etc. These restrictions effectively blocked the transmission of typical respiratory pathogens, and the incidence of related diseases has changed after the outbreak of the COVID-19 pandemic (5). These control measures presumably must have influenced the spread of MP in children. This study aims to understand the status of MP infection in pediatric patients in the context of the COVID-19 pandemic and help to create evidence-based strategies for the early diagnosis and prevention of MP infection. We reviewed the patients who visited the pediatric department of a tertiary hospital in Shandong, China from 2019–2023 with RTI symptoms. A passive agglutination assay was used to test the serum IgM antibodies to MP. By examining the data spanning three periods, i.e., pre-pandemic (January to December 2019), during the pandemic (January 2020 to December 2022), and post-pandemic (January to December 2023), we analyzed the impact of the COVID-19 pandemic on the epidemiological characteristics of MP.

## Methods

### Study subjects

The patient visits at the Department of Pediatrics of Shandong Provincial Third Hospital from January 2019 to December 2023 due to respiratory symptoms were analyzed, including both outpatients and inpatients. For most visits, the clinical diagnosis was upper RTI, pneumonia, bronchitis, bronchopneumonia, tonsilitis, mycoplasma pneumoniae pneumonia etc. The demographic characteristics, clinical information, and MP antibody titer results were summarized. The Inclusion criteria included (1) specimens collected from patients with symptoms of upper respiratory tract infections, (2) Repeated tests within three months only recorded once. The exclusion criteria of this study were as follows: (1) children with malignant tumors or congenital pulmonary airway obstruction; and (2) children with a recurrent chronic respiratory infection. Patients with congenital heart or lung disease, or with immunosuppression, or had received the immunosuppressive therapy, and (3) Patients with incomplete medical records or missing M. pneumoniae test results.

### Detection of *M. Pneumoniae*

The serum antibodies against MP were detected using a passive agglutination kit (Fujirebio, Tokyo, Japan) according to the manufacturer's instructions. MP infection was confirmed if the titer of the serum MP antibody was at least 1:160 (6).

### Statistical analysis

All data were analyzed using SPSS 20.0 (IBM Corp., Armonk, NY, USA). Categorical data, reported as ratios or counts and frequency, were analyzed using the Chi-squared test or Fisher's exact test. Differences were deemed statistically significant when *P* < 0.05.

## Results

### Breakdown of patient visits by year

**Figure 1:**
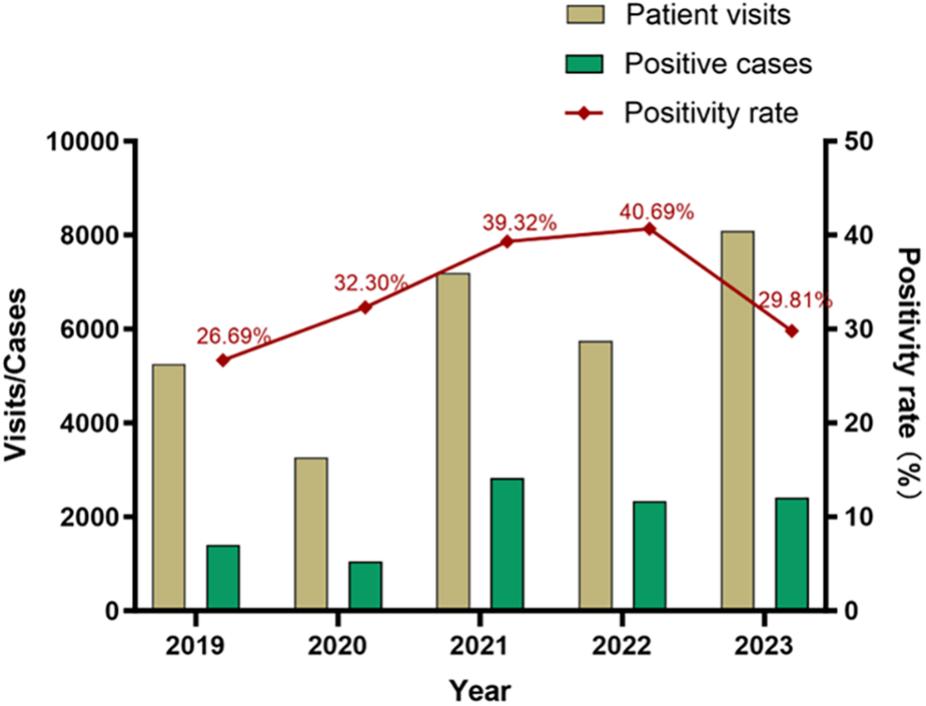
Patient visits, positive cases, and positivity rate of *M. pneumoniae* infection in children.

Table 1 summarizes the visits. From January 2019 to December 2023, there were 29,558 visits to the pediatric department due to RTI symptoms, 10,039 of which involved MP infection. The cumulative positivity rate over the five years was 33.96%. In 2020 (the early stage of the COVID-19 pandemic), there were 3,296 visits that included 1,056 MP positive cases, both of which were significantly lower than the corresponding numbers of the other years. The MP infection peaked in 2021 and 2023. There were 7,196 visits and 2,830 MP positive cases in 2021, and 8,092 visits and 2,412 positive cases in 2023. The positivity rate of MP was high during the later stage of the pandemic (Figure 1), reaching 39.3% (2,830/7,196) in 2021 and 40.70% (2,339/5,749) in 2022, and there was no statistically significant difference between these two years. The positivity rate was the lowest in 2019 (26.7%, 1,402/5,252). The interannual changes of the positivity rate was statistically significantly (*χ*^2^ = 398.1, *P* < 0.001).

**Table 1:** Overview of patient visits and MP cases.

Visits	Total^a^	MP cases^b^	*χ* ^2^ ^c^	*P* value^c^
Five-year total	29,558	10,039		
Breakdown by				
Year			398.1	<0.001
2019	5,252 (17.8%)	1,402 (26.7%)		
2020	3,269 (11.1%)	1,056 (32.3%)		
2021	7,196 (24.3%)	2,830 (39.3%)		
2022	5,749 (19.4%)	2,339 (40.7%)		
2023	8,092 (27.4%)	2,412 (29.8%)		
Age (years)			1,654.5	<0.001
<1	1,582 (5.4%)	66 (4.2%)		
1–2	4,544 (15.4%)	981 (21.6%)		
3–5	11,936 (40.4%)	3,758 (31.5%)		
6–15	11,474 (38.8%)	5,234 (45.5%)		
Month			347	<0.001
Jan	2,759 (9.3%)	958 (34.7%)		
Feb	1,069 (3.6%)	309 (28.9%)		
Mar	1,644 (5.6%)	515 (31.3%)		
Apr	1,453 (4.9%)	487 (33.5%)		
May	1,643 (5.6%)	458 (27.9%)		
Jun	1,812 (6.1%)	588 (32.5%)		
Jul	1,719 (5.8%)	412 (24.0%)		
Aug	1,765 (6.0%)	474 (26.9%)		
Sep	2,644 (8.9%)	801 (30.3%)		
Oct	3,474 (11.8%)	1,272 (36.6%)		
Nov	5,001 (16.9%)	1,837 (36.7%)		
Dec	4,575 (15.5%)	1,928 (42.1%)		
Sex			316.4	<0.001
Male	16,205 (54.8%)	4,783 (29.5%)		
Female	13,353 (45.2%)	5,256 (39.4%)		

^a^ The percentage is calculated relative to the five-year total (*n* = 29,558).

^b^ The percentage is calculated relative to the total number of cases on the respective line.

^c^ Tests if the variation of the positivity rate with each group is statistically significant.

### Seasonality

**Figure 2:**
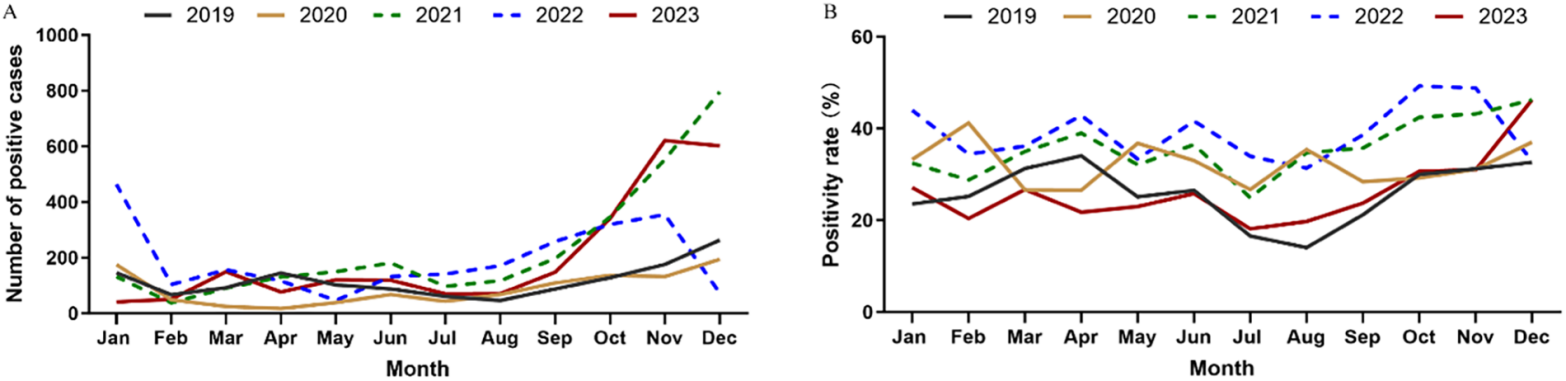
Breakdown of **(A)** positive cases and **(B)** positivity rate of MP infection by month.

In general, for each year, the number of positive cases started to rise in September and stayed at a high level until the next January. However, there was no obvious seasonal pattern in 2020, as the MP infection cases remained at a low level throughout the year. In addition, there was a steep drop of MP infection cases in December in 2022 (Figure 2).

### Difference between age groups

**Figure 3:**
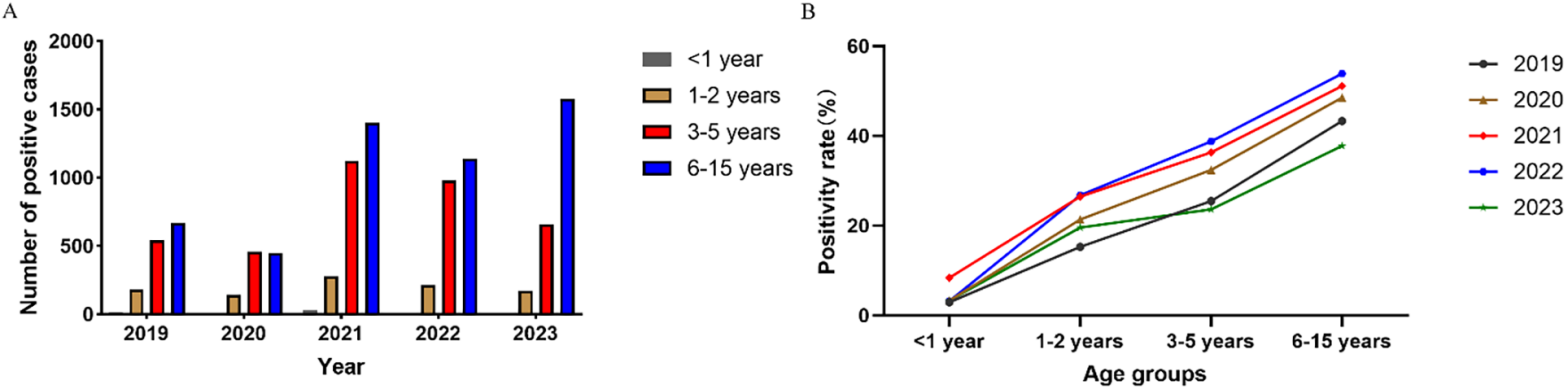
Breakdown of **(A)** positive cases and **(B)** positivity rate of MP infection by age group.

Patients were divided into four age groups: infants (<1 year old), toddlers (1–2 years old), preschoolers (3–5 years old), and students (6–15 years old). Compared to 2019, the cases of MP infection in 2020 decreased for all age groups (Figure 3A). Over the five years, the positivity rate was the highest for students (45.5%, 5,234/11,474) and increased steadily with age (Figure 3B).

### Sex differences

**Figure 4:**
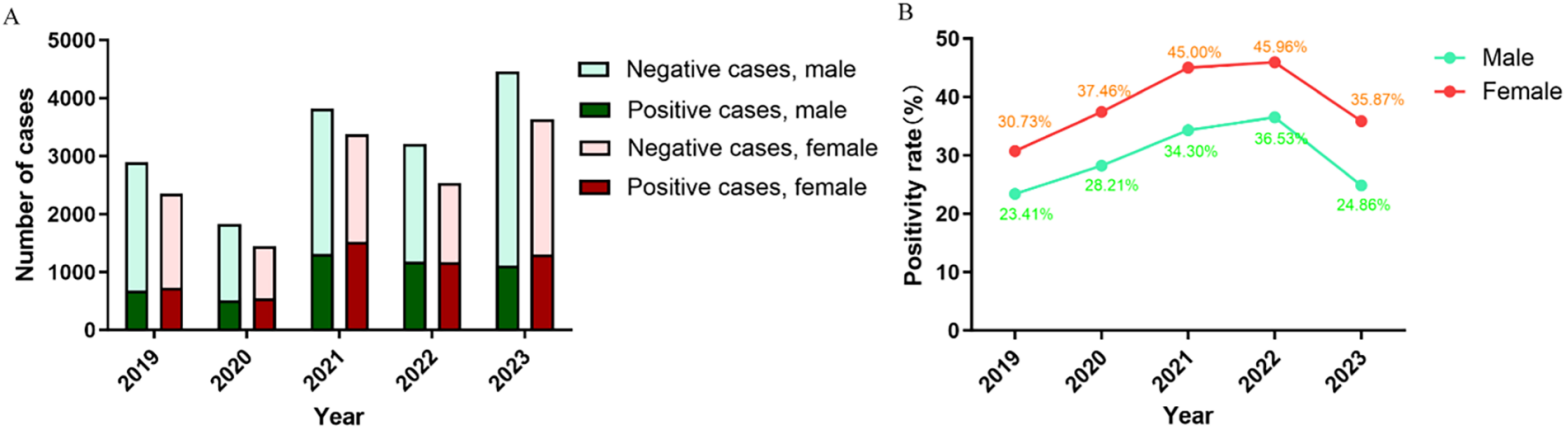
Breakdown of **(A)** positive cases and **(B)** positivity rate of MP infection by sex.

Of the 29,558 visits included in this study, male and female patients accounted for 16,205 (54.8%) and 13,353 (45.2%), and the sex ratio was 1.21:1 (Table 1). In each year there were always more male patients than female patients (Figure 4A), but male patients always had a significantly lower positivity rate (Figure 4B).

### Manifestation

**Figure 5:**
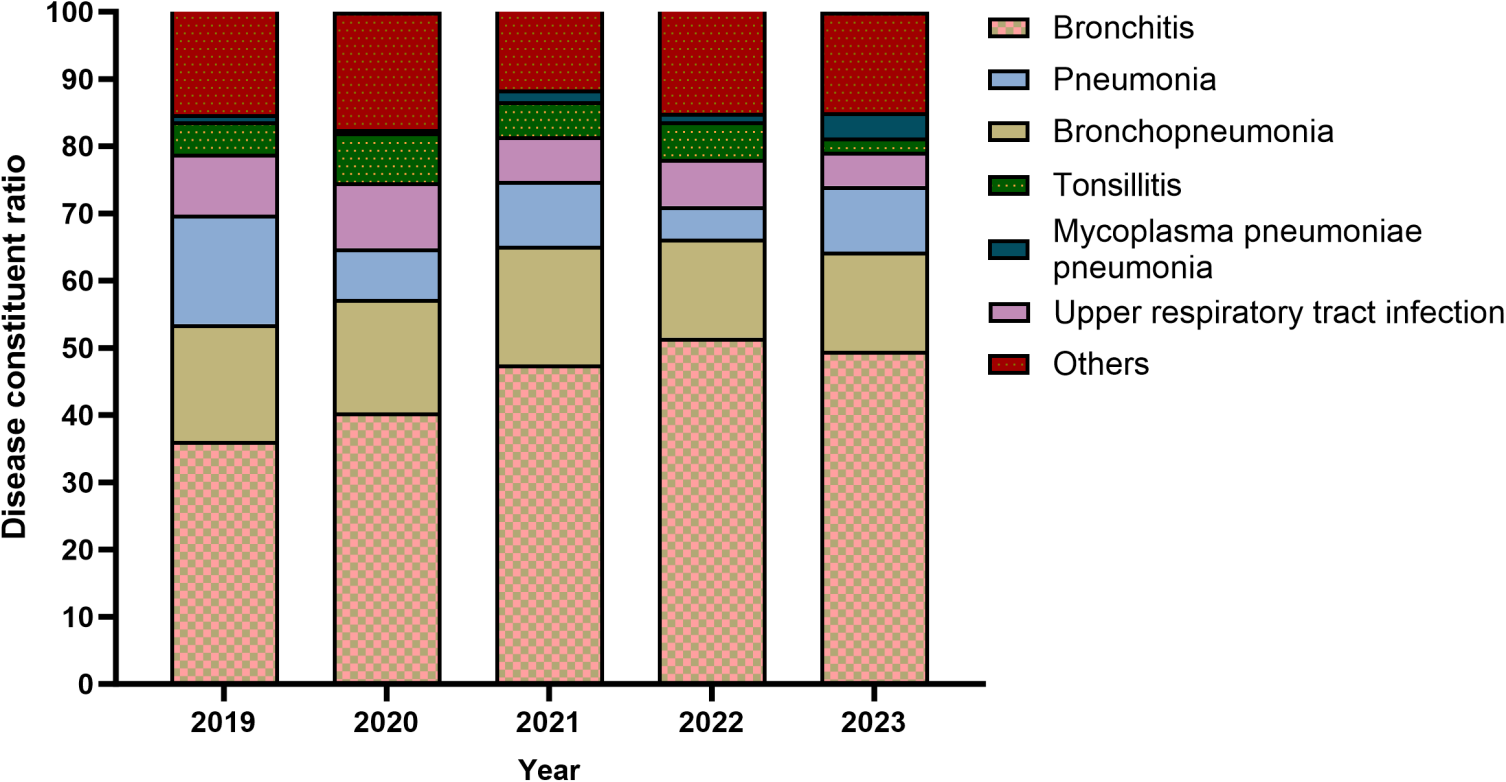
The manifestation of MP infection.

From 2019–2023, the ratio of bronchopneumonia and upper RTI in the manifestation of MP infection decreased steadily, (Figure 5). For the five years in aggregate, bronchitis was the most common manifestation of MP infection.

## Discussion

*M. pneumoniae* is mainly transmitted through the respiratory tract (7), and it is a common cause of acute RTI in children, especially in school-age children. Most infections manifest as mild respiratory illness, but some patients develop severe pneumonia as well as extrapulmonary diseases and complications such as encephalitis, nephritis, myocarditis, etc. (2).

China adopted a series of public health measures since the outbreak of COVID-19, which helped to contain the pandemic. Researchers found that those non-pharmaceutical interventions not only limited the occurrence of COVID-19 but also significantly reduced the transmission of other respiratory pathogens, including influenza virus (8), respiratory syncytial virus (RSV), adenovirus (9), etc. The prevalence of MP infection varies greatly in different regions. In northern China, represented by Beijing, the incidence of MP infection in children is 37.5% and an epidemic peak occurs every 2–3 years (10). In the city of Xi'an in western China, the positivity rate of MP infection in children with RTI symptoms is 26.98%, and a peak occurs every 3–4 years (11). However, in Zhejiang, an eastern coastal province of China, a survey shows that the positivity rate of MP infection among children with RTI symptoms is only 12.2% (12). The different results may be affected by the research samples and detection methods. This study aimed to analyze the changes in the epidemiological characteristics of MP infection in children in east China before and after the COVID-19 pandemic. The results showed that in Shandong, from 2019–2023, the positivity rate was 33.96% in aggregate and there were two peak years (i.e., 2021 and 2023).

In mainland China, unprecedentedly strict measures were put in place since January 2020, including community shutdowns, traffic closures, border controls, and teleworking. These non-pharmaceutical interventions not only limited the spread of SARS-CoV-2 but also affected other diseases significantly (13). In this work, we found that that both the patient visits and the number of positive MP infection cases were significantly lower in 2020 than in other years, which was consistent with the findings of other scholars (14–16). It can be inferred that the strict control measures effectively prevented the spread of MP. However, we found that the positivity rate was higher in 2020 compared to 2019, despite the drop of the case count, although most studies showed that the infection rate of MP dropped sharply in the early stages of the COVID-19 pandemic (16–21).A study of the pediatric patients in Xi'an from 2017–2020 also shows that the positivity rate is higher in 2020 (28.40%, 2,971/10,461) than in 2019 (24.13%, 4,888/20,261) (11). The difference may be relevant to the detection methods and the inclusion standards of each study. In our work, the decline of positive cases identified in 2020 was accompanied by fewer hospital visits. During the pandemic, discretionary visits to the hospital, which is a high-risk area for COVID-19 where people gather, are usually avoided. In addition, a person needs to, by default, obtain a negative PCR test result before visiting a hospital. When patients experience mild RTI symptoms, they often seek medical advice online or resort to simple, empirical treatments at home. Because of this change in behavior, the patients visiting hospitals tend to have a higher positivity rate of RTI pathogens. In fact, we found that the positivity rate of MP infection continued to climb as the pandemic moved forward and dropped only in 2023 after all control measures were eliminated.

In 2021, compared to 2019, the number of pediatric visits increased by 37% (7,196 vs. 5,252) and the number of positive cases increased by 50% (2,830 vs. 1,402). Presumably, in 2020, with the strict control measures, common pathogens were suppressed, exposure to pathogens was reduced, and an “immune gap” have been reported after prolonged low exposure to microorganisms. characterized by waning humoral immunity (22, 23). In 2021, the control measures were relaxed slightly compared to 2020, and a peak year of MP infection occurred because of the recovery of social activities as people lacking immunity started to gather. Of course, with the increased risk of exposure to other pathogens, there are also many mycoplasma tests that are discovered incidentally when screening for other pathogen infections. China abolished its strict COVID-19 control measures as of January 2023, and it is particularly important to continue to monitor MP infections in the post-COVID-19 era. In the United States, the positivity rate of MP infection is lower during the COVID-19 pandemic, and the positivity rate remains lower than that of the pre-pandemic times although it has started to rebound since September 2023 (4). We found that 2023 was another peak year of MP infection and it had the second highest case count in the study period. The incidence of MP infection started to grow in September and the rising incidence is reported across China (24). In 2023, the number of visits was the highest (8,092), but the positivity rate (29.8%) was lower than that of 2021 (39.3%), partly because after the COVID-19 control measures were terminated at the end of 2022, there were a large-scale outbreak of COVID-19 in China from December 2022 to February 2023 and several small outbreaks in 2023 that occurred intermittently. Meanwhile, because various other respiratory pathogens such as the influenza virus and RSV also had an outbreak in 2023 (20), more patients with RTI symptoms but not MP infection visited the hospital, and the positivity rate of MP in 2023 dropped due to a larger denominator. In addition, certain practices that were encouraged during the COVID-19 era, such as hand hygiene, respiratory etiquette, and physical distancing, may have kept the momentum and reduced the burden of MP infection.

Age is an important factor affecting the distribution of pathogens, and it has been reported that preschool children are more susceptible to MP infection (25, 26). We found that of all pediatric patients with MP infection (1 month to 15 years old), the median age was 6 years and the average age was 6.05 years. The positivity rate of MP infection was the highest for school-age children (6–15 years old). The results agreed with the findings for Beijing and Xi'an (10, 11). School-age children live or study in crowded places, and MP can easily spread among them through close contact and droplets. As a result, they are particularly susceptible to MP infection, and future studies should strengthen the prevention and control of MP infection in school-age children. The positivity rate was the lowest for infants (<1 year old), likely because they have less contact with the outside world and are under the intensive care of their parents. The outbreak of COVID-19 had the same impact on all age groups. We also found significant sex difference in MP infection. Male patients made substantially more visits than female patients (16,205 vs. 13,353) but had a much lower positivity rate (29.5% vs. 39.4%), and the differences were statistically significant (*P* < 0.05). Similar observations have been reported for other regions in China (10–12). The outbreak of COVID-19 did not affect the sex difference in MP infection.

There was obvious seasonality in MP infection. A study found that the MP infection in Beijing peaked in the fall (10), and a survey of Xi'an showed that the peak infection was in the winter, followed by the fall (11). In southern China, the outbreaks of MP infection tend to occur in summer or early fall, and for the Zhejiang province the positivity rate of MP infection is high from July to October (12). A study from the United States found that the rate of MP infection is the highest from August to November (27). In this work, we found that in Shandong, the positive MP infection cases concentrated in the fall and winter, and the number of positive cases was at a high level from September to the next January. Thus, effective prevention and control measures should be implemented in fall and winter. We also found that the infection rate of MP was relatively low each year during the winter vacation (February) and the summer vacation (July to August), likely because the transmission was less severe when students left the crowded school environment. The seasonality of MP infection in 2020 was different from that of other years. The Shandong province launched the top-level emergency response to COVID-19 in January 2020. In February 2020, the hospital visits due to RTI symptoms dropped drastically, but the positivity rate of MP increased to 41.2%, likely because the decline of total visits outpaced the decline of MP infection cases. For the rest of 2020, the number of MP cases remained at a low level, which is consistent with the previous research findings of Chengdu, China (26). However, the positivity rate of MP infection fluctuated, likely because the visits were heavily influenced by the adjustments in the COVID-19 control policies. The observed MP infection fell sharply in December 2022, likely because the MP infection was masked by the massive outbreak of COVID-19 upon the cancellation of the control measures.

Bronchitis was the most common among children with MP infection, the ratio of bronchitis in the manifestation of MP infection increased steadily, whereas the ratio of upper RTI decreased substantially. Presumably, the full outbreak of COVID-19 at the end of 2022 damaged the respiratory mucosa in many children, which changed their body's response to MP infection. In Chengdu, bronchopneumonia has the highest proportion (28).

This study has some limitations. First, it is a single-center study. Our findings may not be applicable to other geographic areas, as the prevalence of MP differs regionally due to climate, economic development, lifestyle, etc. Second, the diagnosis of MP infection was only based on the clinical manifestations of RTI and the acute-phase serum antibody titer by one passive agglutination assay. With a single test, the accuracy of the diagnosis is not error-proof, since the result of the IgM assay may be affected by the time and duration of antibody production. Paired serum samples are difficult to obtain from children. Polymerase chain reaction (PCR) detection of RNA or DNA is the most sensitive (29), but it is not routinely used for MP screening due to its high cost. Third, the survivorship bias must be considered in interpreting the results, as the findings at a large tertiary hospital may not accurately reflect the epidemiology in the broader community. Fourth, the included samples had a certain selection bias, these data do not include patients who have not been tested for M. pneumoniae.

Our study retrospectively analyzed the epidemiological changes of MP infection in patients with RTI symptoms before, during, and after the COVID-19 pandemic in Shandong, China. The positivity rate of MP infection varied notably from year to year, and there was also obvious seasonality in the prevalence of MP. In addition, patients of different sexes and within different age brackets had varying positivity rates. In the early stage of the pandemic, with strict intervention measures, the spread of MP was suppressed, the number of positive cases dropped significantly, but the positivity rate increased. In the middle and late stages of the pandemic, the control measures were more flexible, and there were two peak years of MP infection. Compared to 2019 (e.g., pre-pandemic), in 2023 (e.g., post-pandemic), among the MP cases, the proportion of bronchitis increased steadily, and the proportion of upper RTI decreased significantly. More epidemiological surveillance of MP is necessary to better understand and manage the changing dynamics and improve the early prevention measures. In future studies, we plan to prospectively monitor patients with acute respiratory infections of all ages through a combination of serological testing and nucleic acid testing. Reveal the statistical relationship between pathogens, and provide a direction for exploring the complex interactions between viruses and viruses and bacteria. The results of this study provide a reference for the surveillance and prevention of viral and bacterial RTI in eastern China in the post-pandemic era.

## Data Availability

The original contributions presented in the study are included in the article/Supplementary Material, further inquiries can be directed to the corresponding authors.
